# Seasonal and age effects on *in vitro* embryo production in domestic cats under a standardized protocol

**DOI:** 10.3389/fvets.2025.1647305

**Published:** 2025-08-19

**Authors:** Andrea Priego-González, Ana Munoz-Maceda, Manuel Fuertes-Recuero, Paula Medranda Ayjon, María Aránzazu Sánchez-Calabuig, Sandra Barroso-Arévalo, María Jesús Sánchez-Calabuig

**Affiliations:** ^1^Department of Medicine and Surgery, Faculty of Veterinary Medicine, University Complutense of Madrid, Madrid, Spain; ^2^Complutense Veterinary Teaching Hospital, University Complutense of Madrid, Madrid, Spain; ^3^Department of Physiology, Faculty of Veterinary Medicine, University Complutense of Madrid, Madrid, Spain; ^4^Faculty of Health Sciences, HM Hospitals, University Camilo José Cela, Madrid, Spain; ^5^Instituto de Investigación Sanitaria, HM Hospitales, Madrid, Spain

**Keywords:** domestic cat, *in vitro* embryo production, assisted reproductive technologies, seasonality, *Felis catus*

## Abstract

**Introduction:**

*In vitro* embryo production (IVP) in the domestic cat (*Felis catus*) remains highly variable owing to intrinsic reproductive traits and the absence of fully standardized protocols.

**Methods:**

We retrospectively analysed 108 IVP replicates produced under a single protocol (2020–2024) to quantify the effects of season, donor age and methodological parameters on oocyte yield and embryo development.

**Results:**

Winter proved the most favourable season for both oocyte recovery and blastocyst formation, whereas spring, despite lower initial yields, achieved the greatest post-selection oocyte retention. Donor age correlated negatively with oocyte number; however, older queens showed higher blastocyst conversion rates, suggesting that only developmentally competent oocytes persist at advanced age.

**Discussion:**

These results highlight the need for rigorous donor selection and season-tailored IVP protocols to enhance embryo yield, quality and dataset reproducibility, prerequisites for deciphering embryo–maternal signalling mechanisms. Because the domestic cat serves as a valuable translational model for endangered felids, optimizing these factors will advance both feline ART and conservation breeding programs.

## 1 Introduction

Assisted reproductive technologies (ARTs) have evolved into powerful tools for studying early conceptus-maternal interactions. In the domestic cat (*Felis catus*), a seasonally polyoestrous, induced-ovulating species, ARTs include artificial insemination (1), embryo transfer (2), IVF, and cryopreservation of gametes and gonadal tissues (3). Nevertheless, clinical uptake is still dominated by sperm cryopreservation and artificial insemination, whereas IVF-derived procedures remain limited in clinical practice despite the growing interest in feline IVF (4, 5).

This limited application is partly rooted in unresolved biology. Felids lack a defined luteotrophic signal for maternal recognition of pregnancy (6). Consequently, endocrine and immune events at implantation remains poorly characterized (7, 8). Targeted retrieval of oocytes for *in vitro* embryo production (IVP), together with proteomic and transcriptomic analyses of follicular and uterine fluids, now offers unprecedented access to otherwise hidden peri-implantation stages. Recent studies show that feline blastocysts secrete annexins, heat-shock proteins and metabolic enzymes. This stage-dependent release confirms that the embryo actively modulates its uterine environment (9). Extracellular vesicles (EVs) are also key mediators in this dialogue. For example, oviductal EVs bind feline sperm and enhance its motility and fertilizing capacity (9). However, *in vivo* data on embryo- or endometrium-derived EVs during early feline pregnancy are still lacking.

Comparative studies in livestock help to bridge the current knowledge gap. In cattle, the embryo alters oviductal and uterine gene expression within 3 days after oestrus, dampening local immune pathways (10). Supplementing bovine IVF media with extracellular vesicles or single growth factors such as IGF-1 partly corrects culture-induced transcriptomic deficits, and microfluidic “oviduct-on-a-chip” systems reproduce these benefits *in vitro* (11). In pigs, IGF-2 and CXCL12 promote conceptus elongation, whereas in horses, uterine secretions rich in osteopontin (SPP1), MFGE8 and IGFBP1 facilitate adhesion and nutrient exchange (12). Together, these examples illustrate a conserved interplay of secreted signals, physical cues and cellular responses that orchestrates early embryo–maternal communication.

**Figure 1:**
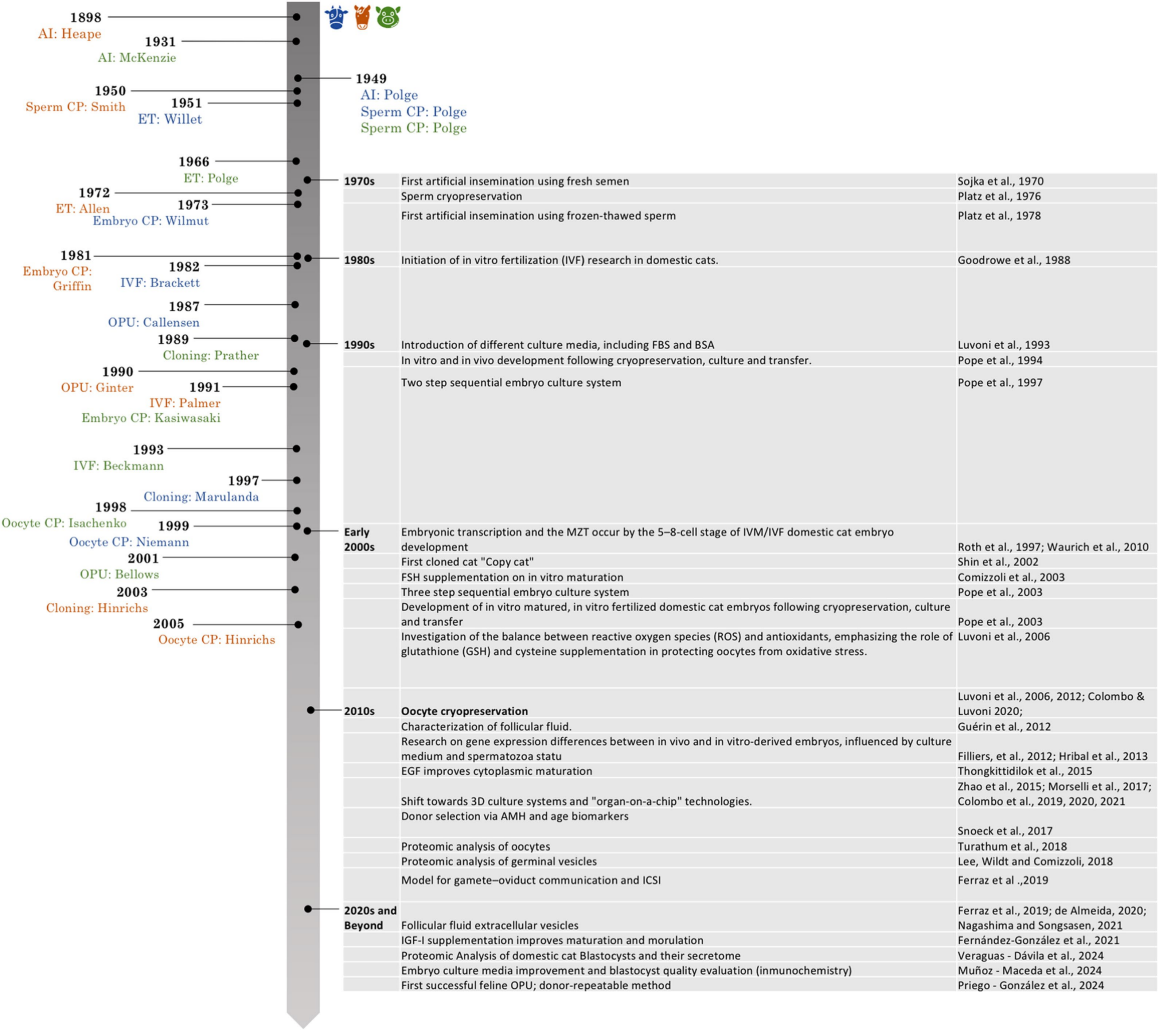
Historical timeline of key milestones in assisted reproductive technologies in bovine, porcine and equine species, with emphasis on developments in the domestic cat. Icons: Flaticon.com.

Milestones in ART in livestock species were progressively achieved throughout the 20th century. Artificial insemination was first reported in bovine and equine species at the beginning of the century, followed shortly thereafter in swine. Sperm cryopreservation was successfully established in all three species around the 1950s. Embryo cryopreservation followed, with initial successes reported in bovine in the early 1970s, equine in the 1980s, and porcine in the 1990s. Ovum pick-up (OPU) techniques were first described in horses in 1987, followed by cattle in 1990 and pigs in 2001 (Figure 1). Despite these advances, the application of ARTs in felids remains less efficient compared to domestic species such as cattle or swine (13) (Figure 1). Feline semen typically exhibits poor quality, with high levels of morphological abnormalities and low motility (3, 14). On the female side, challenges include seasonal reproductive patterns, anatomical limitations that hinder oocyte retrieval and embryo transfer (ET), and suboptimal IVP rates due to inefficiencies in *in vitro* maturation (IVM), fertilization (IVF), and embryo culture (15, 16), and trans-abdominal OPU in queens (2).

Given the limited availability and high value of biological samples from wild felids, the domestic cat has emerged as a valuable model for optimizing ART protocols. Routine gonadectomies provide access to a steady supply of gametes, enabling refinement of techniques that can later be adapted to species-specific requirements in wild felids (17, 18). However, achieving optimal IVP conditions in domestic cats remains a major hurdle. Recent reports document IVM success rates of approximately 50–60% (19) and blastocyst yields of only 10–20% of cultured zygotes (20).

In the Northern Hemisphere, domestic cats exhibit seasonal polyestrous reproductive patterns, with the breeding season typically beginning in January or February, peaking in February and March, and concluding between June and November (21). Efforts to emulate *in vivo* physiological conditions *in vitro* have focused on both biochemical and physical aspects of the female reproductive tract. Strategies include supplementation with proteins, fatty acids, or hormones; modulation of incubation times; media changes or renewal; and the use of proteomic and metabolomic analyses to enrich culture media (16). Physical factors such as co-culture systems, oocyte or zygote denudation, drop volume adjustments, and three-dimensional culture models are also under investigation.

Many researchers have specifically addressed the optimization of *in vitro* culture (IVC) conditions to improve embryo development rates in the domestic cat. Studies have examined the use of SOF medium (22), as well as the impact of fetal bovine serum (FBS) and bovine serum albumin (BSA) supplementation (23, 24). Adaptations of IVC media from bovine and human ART have also been successfully tested in feline systems (24–26), yielding cleavage rates between 53 and 74% and blastocyst rates from 13 to 20%, depending on the protocol. IVC media management strategies vary, with some researchers employing a one-step approach without medium change (27), while others perform multiple medium renewals, either complete (23, 24, 28) or partial (25), to better mimic the dynamic environment of the queen’s reproductive tract.

These protocols have been evaluated not only by cleavage and blastocyst rates or timing of blastocyst formation (24, 28, 29), but also through immunostaining, cell count analysis (23, 24, 29), and gene expression profiling via RT-qPCR (30–32). Our IVP results support the potential of 3D culture systems (with FBS supplementation, with a combination of BSA and FBS, and a human IVC commercial medium), with the BSA-FBS group achieving the highest (though not statistically significant) blastocyst rates: 20.48% ± 7.99 of cultured zygotes, and 36.31% ± 15.77 of cleaved embryos. Moreover, quality results supported a potential benefit of the BSA-FBS combination to the quality of domestic cat blastocysts (28).

Several critical gaps currently limit the reproducibility of feline IVP and impede, therefore, progress in decoding embryo-maternal signalling pathways. Protocols for oocyte retrieval vary widely among laboratories, ranging from post-sterilization collection to OPU, without consensus regarding the optimal timing of collection relative to the natural ovarian cycle (2, 16). Most protocols implicitly assume the use of immature oocytes; however, since ovaries are typically collected post-ovariectomy and the reproductive status of the donor is rarely documented, the precise physiological stage of the female and consequently, the true developmental stage of the recovered oocytes, often remains unknown (19, 33). In addition, IVM conditions, culture media compositions, and fertilization methods vary significantly across studies, complicating the evaluation of true biological variation. Similarly problematic is the lack of standardized schedules for collecting oviductal and uterine fluids. Without synchronising sample acquisition to specific peri-ovulatory or peri-implantation windows, it is impossible to compare the repertoire of cytokines, growth factors, extracellular vesicles, or other signalling molecules across studies. Finally, donor-selection criteria (such as age, parity, season, and circulating anti-mullerian hormone levels) remain inconsistent, despite clear influences on oocyte competence and downstream embryo development. To overcome these obstacles and advance our understanding of embryo–maternal communication in the domestic cat, we must first establish a comprehensive set of guidelines that specify (i) the optimal reproductive stage and seasonal window gamete collection, (ii) precise protocols for sample handling (including optimal media, centrifugation speeds, filtration steps, and storage duration and temperatures), (iii) potential modifications during IVP protocols that may play a crucial role in compensate possible detrimental effects of age of the donor and season, and (iv) standardized donor-selection protocols for IVP that maximise both embryo yield and quality. Only through such harmonisation can we generate comparable datasets, across laboratories and geographies, and thereby decode the molecular dialogue that underpins successful feline implantation. The findings aim to provide a robust foundation for the refinement and standardization of IVF protocols in this species and to serve as a starting point for future investigations into embryo–maternal communication.

## 2 Materials and methods

This section comprises two main components: (1) the protocols for oocyte recovery, IVM, fertilization (IVF) and culture (IVC) as originally described by Munoz-Maceda et al. (28), and a retrospective evaluation of domestic-cat embryo-production records, encompassing season, donor age, hormonal status and oocyte/embryo outcomes (2), in order to identify sources of variability and propose standardized guidelines.

### 2.1 Animals

**Figure 2:**
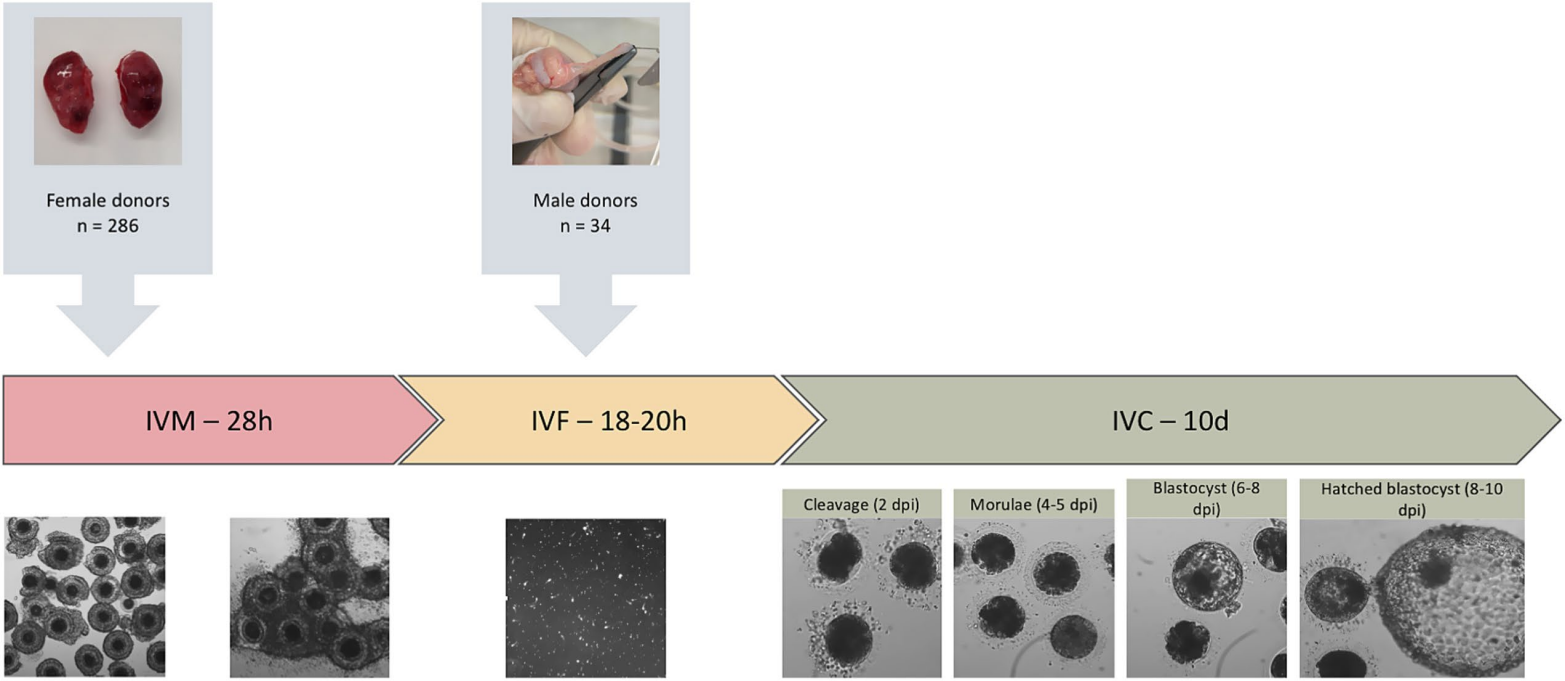
Experimental design of in vitro embryo production in domestic cats. The diagram illustrates the overall workflow, including the number of female oocyte donors and male sperm donors, and the sequential stages of in vitro maturation (IVM), in vitro fertilization (IVF), and in vitro culture (IVC), during which embryo development was evaluated based on cleavage, morula, and blastocyst rates.

A total of 320 domestic cats (*Felis catus*), comprising 286 female oocyte donors and 34 male sperm donors, ranging in age from 6 months to 8 years, were included in this study (Figure 2). Gonads were collected and transported within 3 hours post-surgery in 0.9% saline solution to the Reproduction Laboratory at the Complutense University of Madrid (HCVC) for subsequent processing. During transportation, ovaries were maintained at 30°C, while testes were kept at room temperature. All procedures adhered to Spanish Animal Protection Regulation RD53/2013, in accordance with European Union Directive 2010/63.

### 2.2 Oocyte recovery

Following excision, each ovary was placed immediately into pre-warmed sterile saline solution (0.9% NaCl), and maintained at 30°C. Under sterile conditions, oocytes were recovered by slicing in HEPES-TCM-199 (Sigma Aldrich, St Louis, MO, United States). Briefly, the ovarian cortex was incised with a scalpel and gentle mechanical pressure was applied to the cut surface to expel follicular fluid and release cumulus–oocyte complexes (COCs). Recovered COCs (*n* = 13,912 total) from all queens in a replicate were collected together under a stereomicroscope, washed in HEPES-TCM-199 (Sigma Aldrich), and graded according to cumulus-cell compactness and ooplasm homogeneity. Only grade I and II oocytes (*n* = 6,285 total) were selected for subsequent IVM.

### 2.3 *In vitro* maturation

Selected COCs (*n* = 6,285) were washed four times in maturation medium, TCM-199 (Earle’s salts, Gibco) supplemented with 25 mM NaHCO₃, 1.12 mM L-cysteine, 2.2 mM sodium lactate, 0.36 mM sodium pyruvate, 0.4% BSA, and 25 ng/mL epidermal growth factor (EGF; Sigma-Aldrich), and distributed into groups of 30–50 oocytes. Each group was placed in a four-well plate, in 500 μL of the same medium and incubated for 28 h at 38.5°C in a controlled atmosphere of 20% O₂ and 5% CO₂.

### 2.4 Sperm preparation and *in vitro* fertilization

Epididymal samples were collected from the cauda epididymis testes obtained from 34 tomcats (1–6 years old). Spermatozoa were retrieved by a modified retrograde-flushing technique as previously described (28). Briefly, the free end of the ductus deferens was cannulated with a blunted 27 G butterfly needle connected to a 1 mL syringe pre-loaded with cryopreservation extender (Caniplus Freeze, Minitube^®^, Tiefenbach, Germany supplemented with 20% egg yolk). The extender was then slowly infused until the cauda epididymidis was visibly distended. Finally, an incision was performed in the cauda with a sterile scalpel, allowing the sperm-rich fluid to be collected directly into a sterile Eppendorf. Following initial evaluation, samples were adjusted to a final concentration of 40 × 10^6^ spermatozoa/mL with the same cryopreservation extender. Sample was placed in a water bath and gradually cooled to 4°C for 90 min. Aliquots were loaded into 250 μL straws and sealed with a thermosealer (Minitube^®^). Then, straws were placed horizontally in liquid nitrogen (LN2) vapors for 10 min min at a height of 7 cm. Lastly, they were directly plunged in LN2 until analysis.

Cryopreserved epididymal spermatozoa, retrieved post-surgery from healthy toms, were thawed by immersion of 0.25 mL straws in a 37°C water bath for 50 s. The thawed suspension was layered onto a single-layer density gradient (80% Bovipure^®^ over 20% Bovidilute^®^; Nidacon, Molnan, Sweden) and centrifuged at 300 × g for 8 min. The sperm pellet was washed in Boviwash^®^ (Nidacon) by centrifugation at 300 × g for 5 min, then resuspended in FERT-TALP medium (Tyrode’s supplemented with 25 mM NaHCO₃, 22 mM sodium lactate, 1 mM sodium pyruvate, 46 mg/mL fatty acid–free BSA, and 10 μg/mL heparin, Sigma-Aldrich). Immediately prior to IVF, thawed semen was evaluated with a computer-assisted sperm-analysis (CASA) system (Hamilton Thorne IVOS II). Only samples displaying ≥60% progressive motility and ≥70% total motility were used for insemination. Final sperm concentration was adjusted to 1 × 10^6^ spermatozoa/mL. Groups of mature oocytes were co-incubated with sperm in a four-well plate, at 38.5°C for 18–20 h under 20% O₂ and 5% CO₂. After co-incubation, degenerated presumptive zygotes were discarded, and morphologically normal presumptive zygotes (*n* = 4,546) proceeded to culture.

### 2.5 *In vitro* culture

Presumptive zygotes were washed four times in laboratory-made synthetic oviductal fluid (SOF) supplemented with 3 mg/mL BSA. 18 to 25 embryos were placed in each 25 μL SOF droplets under mineral oil and cultured at 38.5°C in a gas mixture of 5% O₂, 5% CO₂, and 90% N₂ for 10 days. The culture medium was refreshed on Day 4 post-insemination (dpi) by replacing it with SOF containing 10% fetal bovine serum (FBS). Cleavage rates were recorded at 4 dpi, and blastocyst formation and hatching were assessed daily from 6 dpi through 10 dpi (23, 28). Embryos were cultured until Day 10 to capture the full spectrum of in-vitro developmental kinetics in cats; under these conditions, complete expansion and hatching typically occur between Days 8 and 10 (29). Hatching was recorded because it represents the most advanced functional indicator of implantation competence available *in vitro* (Figure 2).

### 2.6 Retrospective analysis of seasonality, donor age, and reproductive variables

To evaluate sources of variability and inform standardization, data from 108 replicates conducted between January 2020 and June 2024 were extracted from our laboratory’s database. For each cycle, the following parameters were recorded: Date of oocyte retrieval [categorized by month and season: spring (March–May), summer (June–August), autumn (September–November), winter (December–February)]; Donor age [categorized in young (less than 2 years); adult (2–8 years) and geriatric (more than 8 years)]; Oocyte yield (total recovered); selected oocytes; blastocyst per zygote; and blastocyst per cleaved embryos.

### 2.7 Statistical analysis

All statistical procedures were conducted in IBM SPSS Statistics v.29 (IBM Corp., Armonk, NY, United States); figures were generated from the estimated marginal means in Microsoft Excel 365. The experimental factors of interest were maternal age (three levels: young, adult, geriatric), season (winter, spring, summer, autumn) and calendar month (January–December). Four response variables were evaluated: the number of oocytes recovered per aspiration, the number of oocytes selected for IVM, the proportion of blastocysts per zygote, and the proportion of blastocysts per cleaved embryo. Data distributions were screened with histograms, Q–Q plots and the Shapiro–Wilk test. Homogeneity of variance was assessed with Levene’s test. Percentage variables were arcsine–square-root transformed to stabilise variance; counts did not require transformation. All analyses were carried out on the transformed scale and back-transformed for presentation.

Each outcome was analysed in three separate one-way models, one for each factor (age, season, month). Models were fitted with the UNIANOVA routine, which provides estimated marginal means (EMMs) that correct for unequal sample sizes. When Levene’s test indicated heteroscedasticity (*p* < 0.05), the Brown–Forsythe adjustment was invoked automatically by SPSS. Pairwise differences among factor levels were examined with the Games–Howell test, chosen because it is robust to heteroscedasticity and unequal n. Adjusted *p*-values are reported in Supplementary Table 1. Magnitudes of the main effects were quantified with partial eta squared (η^2^ₚ) and interpreted as small (≈ 0.01), medium (≈ 0.06) or large (≥ 0.14). All *p*-values are two-tailed with an a-level of 0.05. Results are presented as mean ± standard error of the mean (SEM) unless stated otherwise; figures display the back-transformed EMMs with their 95% confidence intervals.

## 3 Results

A total of 569 oocyte-recovery events and 108 complete IVP cycles were conducted from January 2020 through June 2024. Key outcome measures, number of oocytes recovered per queen, selected oocytes for IVM per replicate over the total recovered, cleavage rate (percentage of zygotes cleaving by Day 4), and blastocyst rate (percentage of zygotes forming blastocysts by Day 10), were examined with respect to season, donor age category, and oocyte-recovery method. Seasonal and age-related trends are summarised below (all values are means, and complete numerical summaries are provided in Supplementary Table 1).

### 3.1 Effect of season on embryo development parameters

Season exerted a consistent influence on the reproductive parameters. Oocyte retrieval peaked in winter (mean of 16.95 ± 3.28), falling by ~33% in spring; the small–medium effect (η^2^ ≈ 0.04) suggests environmental conditions in winter favour follicular response (Supplementary Table 2).

**Figure 3:**
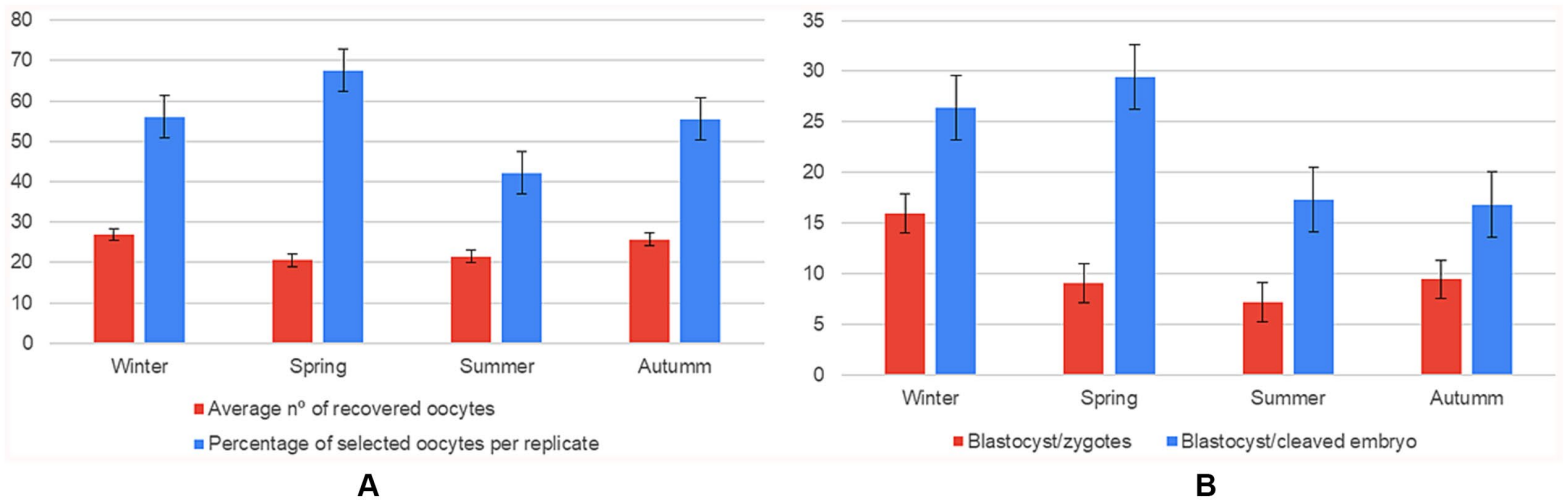
Effect of season on oocyte recovery and in vitro embryo development (*n* = 108 IVP replicates). Six panels showing mean ± SD for: **(A)** average number of oocytes recovered compared to the average number of oocytes selected per replicate; **(B)** number of blastocysts formed per zygote compared to the blastocyst-to-cleaved-embryo ratio, grouped by season (winter, spring, summer, autumn).

Despite lower overall yield, oocytes recovered in spring had higher selection rates, which retained ~40% more oocytes than summer; however, winter still outperformed summer, indicating that lower summer scores are not solely due to stricter selection criteria. A pairwise Games–Howell test showed that spring produced significantly more selected oocytes per replicate than autumn (67.6 ± SE vs. 55.9 ± SE; mean difference ≈ 11.7 oocytes, *p* < 0.01), representing an increase of ≈ 21% and constituting a medium-sized seasonal effect (partial η^2^ ≈ 0.12). Developmental competence showed the strongest seasonal modulation. The proportion of blastocysts per zygote was 1.8-fold higher in winter than in spring/summer (η^2^ ≈ 0.15). When normalised to the number of cleaved embryos, spring and winter remained superior, whereas summer lagged by ~10 percentage points (Figure 3).

### 3.2 Effect of the month

Marked intra-annual fluctuations were detected across all reproductive endpoints, with a pronounced spring trough in April and a late-autumn/early-winter peak in November–December. The number of oocytes recovered fell to its lowest point in April (mean of 15.86 ± 1.90) and increased almost two-fold to reach ≈ 34 in September and December (*F*₁₀, ₄₅₈ = 5.56, *p* < 0.001, η^2^ = 0.108) (Supplementary Table 3).

Although the quantity of oocytes ultimately selected per replicate did not differ statistically among months (*F*₁₀, ₆₉ = 1.71, *p* = 0.095), values were consistently around 50–55 throughout the first half of the year and showed a descriptive jump to ≈ 68 in December.

**Figure 4:**
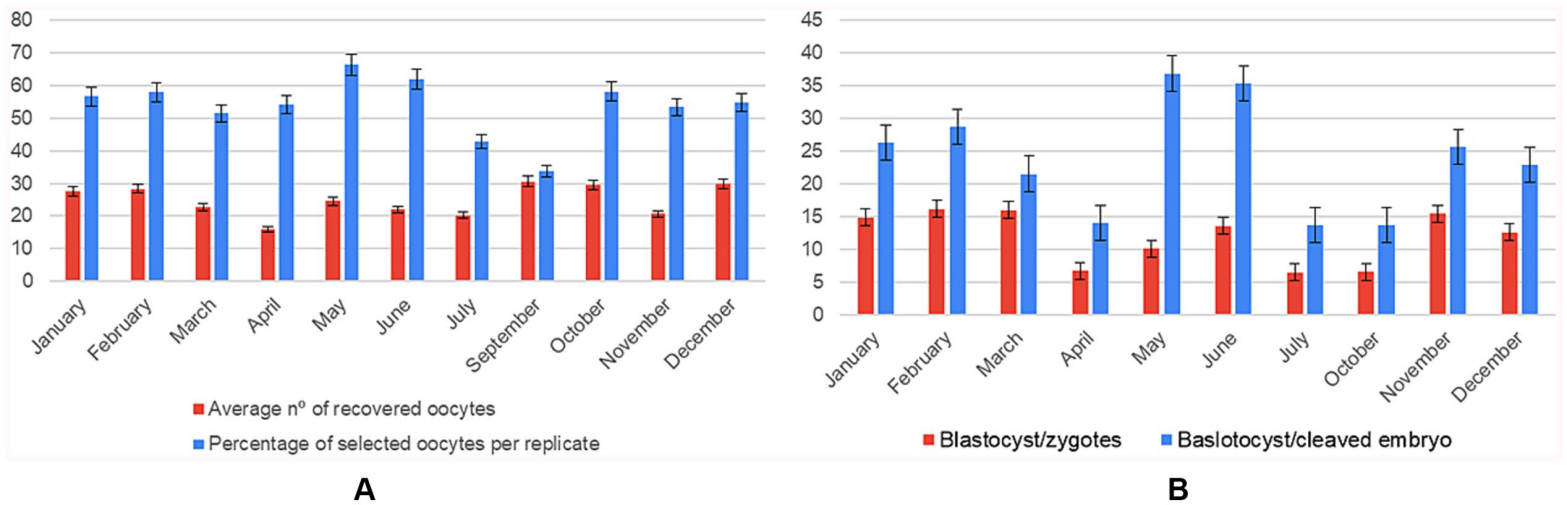
Monthly variation in oocyte recovery and in vitro embryo development (*n* = 108 IVP replicates). Two panels showing mean ± SD for each month (January–December): **(A)** average number of oocytes recovered compared to the average number of oocytes selected per replicate; **(B)** number of blastocysts formed per zygote compared to the blastocyst-to-cleaved-embryo ratio.

Embryo developmental competence exhibited the clearest seasonal imprint (*F*₉, ₃₃₁ = 13.0, *p* < 0.001, η^2^ = 0.261). Efficiency declined from an early-year high in January (~28%) to ≤ 10% in April, then recovered steadily to ~19% by November–December (Supplementary Table 4). The same pattern was amplified when the denominator was limited to cleaved embryos (*F*₉, ₃₂₁ = 22.96, *p* < 0.001, η^2^ = 0.392): conversion rates averaged only ~14% in April–May yet rose to ~45% in December, tripling the spring efficiency (Figure 4).

### 3.3 Effect of the age

A significant age effect was detected for the number of recovered oocytes: young queens yielded 21% more oocytes than adults and 46% more than geriatric counterparts (small-to-medium effect, η^2^ = 0.032). In contrast, the quantity of oocytes selected per replicate did not differ across age groups, indicating that laboratory selection efficiency compensated for the reduced ovarian output in older animals (Supplementary Table 5).

**Figure 5:**
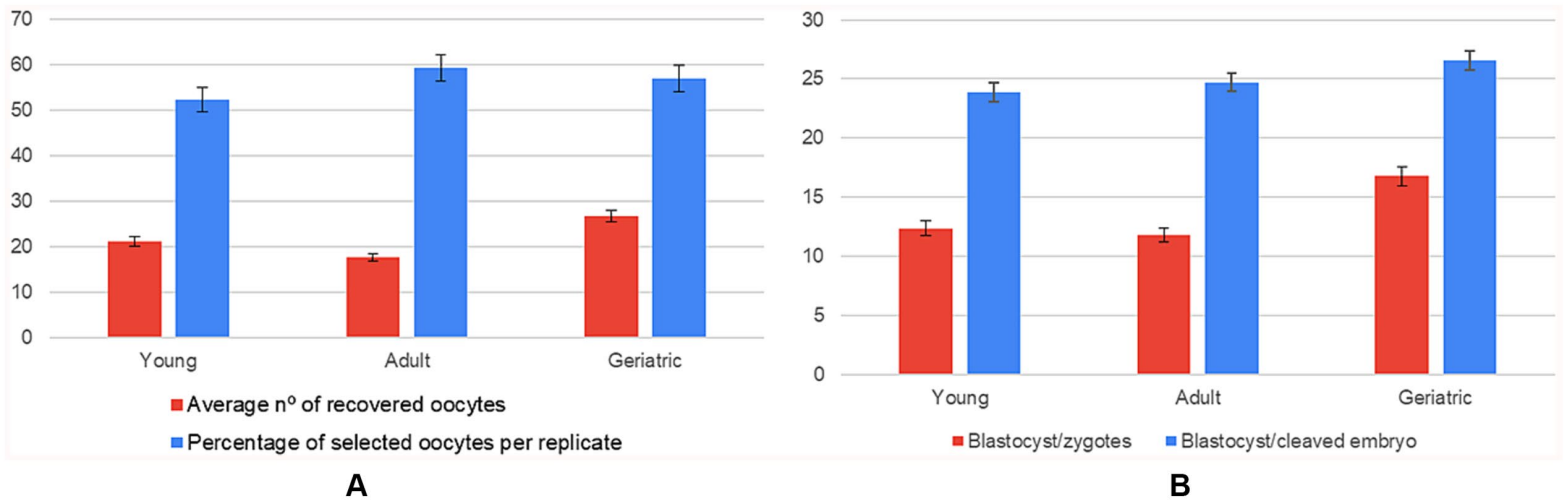
Influence of donor age on oocyte recovery and in vitro embryo development (*n* = 108 IVP replicates). Six panels showing mean ± SD for: **(A)** average number of oocytes recovered compared to the average number of oocytes selected per replicate; **(B)** number of blastocysts formed per zygote compared to the blastocyst-to-cleaved-embryo ratio, grouped by age range (young, adult, geriatric).

Regarding developmental competence, the proportion of blastocysts per zygote increased with age (η^2^ = 0.056). Geriatric queens achieved a 34% relative gain over young and adult groups, suggesting a selective retention of high-quality oocytes. The downstream conversion from cleaved embryo to blastocyst showed a similar upward trend but did not reach statistical significance (*p* = 0.27) (Figure 5; Supplementary Table 6).

## 4 Discussion

This study provides a comprehensive evaluation of seasonal, age-related, and methodological influences on IVP in the domestic cat. The findings offer a robust foundation for the refinement and standardization of IVF protocols for this species, mirroring efforts previously achieved in livestock species. The present analysis is distinguished by its 4-year data set, the simultaneous assessment of season and donor age, and the application of a single, harmonised IVP protocol, thereby minimising inter-laboratory variability. A statistically significant seasonal effect was observed in the number of oocytes recovered per female, with higher yields during winter and autumn compared to spring. Laboratory selection, however, favored spring replicates, which retained approximately 40% more oocytes than summer samples and 21% more than autumn ones. This apparent discrepancy underscores the importance of distinguishing between raw recovery rates and post-selection competence when defining standardized protocols. The increased recovery rate during winter aligns with previous studies reporting higher yields during the breeding season (34). These results reinforce the notion that reproductive seasonality modulates oocyte competence, cytoplasmic maturation and, ultimately, blastocyst output in felids (34, 35). This last study first reported that domestic cat oocytes collected during the non-breeding season exhibited reduced nuclear maturation rates and failed to develop to the blastocyst stage. Subsequent studies across diverse geographic regions and breeding conditions have confirmed this seasonal dependency, attributing it to diminished ovarian activity and impaired follicular development (36). Our data expand these observations by demonstrating that, although non-breeding oocytes remain numerically abundant, their post-selection retention and developmental competence are comparatively poor, highlighting an unmet need for season-specific culture optimization.

Notably, our data revealed a high oocyte yield in autumn, contrasting with previous reports. One plausible explanation is the longer ovary-processing time applied during the non-breeding season (internal data), implemented to offset the smaller follicle size typical of this period. Nevertheless, despite this increase in oocyte recovery in autumn, laboratory selection consistently favored spring replicates over those from the non-breeding season. This suggests that the additional time invested in autumn processing does not translate into higher-quality cohorts and may therefore be economically unjustified. In the analysis of monthly oocyte selection, values remained relatively stable (50–55 oocytes per ovary) throughout the first half of the year, with a marked increase to approximately 68 recovered oocytes in December. Collectively, these findings designate winter as the optimum reproductive window, while spring, although less productive, yields the highest laboratory retention and maintains consistent blastocyst formation.

The exact mechanisms underlying these seasonal differences remain unclear. Proposed explanations include reduced circulating FSH levels and a subsequent seasonal decrease in cat oocyte sensitivity to FSH, which have been observed both *in vivo* and *in vitro* (34). Furthermore, the link to a reduced density of FSH receptors in oocytes or surrounding cumulus cells was studied (37), evidencing that these receptors seemed to be lower outside the breeding season and in immature COCs. Oocytes fertilized during the non-breeding season appeared to have reduced developmental potential; nonetheless, the expression of FSH receptor genes was unrelated to the latter, suggesting more complex mechanisms and factors may contribute to the seasonal effect. Notably, Comizzoli et al. (36) demonstrated that the meiotic and developmental competence of oocytes collected during the non-breeding season is not entirely lost. Rather, limitations in *in vitro* conditions, such as suboptimal hormonal supplementation or insufficient antioxidant support, may constrain the developmental potential of these oocytes. These findings suggest that, although reproductive quiescence poses a physiological challenge to IVM success, it can be at least partially mitigated by optimizing culture conditions, including the use of FSH supplementation and antioxidant additives (38). In contrast, the decline in both oocyte selection rates and embryo quality observed during summer in all studies mentioned included ours, highlights the need for seasonally adapted protocols, as occurs in other species. Adjustments to culture conditions or the incorporation of targeted supplementation strategies may be necessary to sustain optimal IVP efficiency during periods of elevated ambient temperature.

Moreover, in contrast to the findings of Spindler et al. (34), who reported peak blastocyst development between February and April (breeding season in the US) and no blastocyst formation during the non-breeding season, we identified a low-performance window in mid-spring, characterized by reduced ovarian output and diminished embryo quality, and an optimal window spanning November to December, during which both oocyte yield and developmental competence were maximized. While the earlier study reported a complete absence of blastocyst development outside the breeding season, we observed blastocyst production rates ranging from 9 to 16% across all seasons, indicating improved consistency in embryonic development. Several factors may explain this divergence.

First, only Grade I and II oocytes, classified according to the criteria established by Wood et al. (39) were selected for IVP in our study, ensuring higher initial quality. Second, significant advancements in IVP media and protocols over the past two decades have likely enhanced both cytoplasmic and nuclear maturation, contributing to more uniform developmental outcomes throughout the year. Third, our oocytes were matured for 26–28 h, compared to 32 h in the earlier study, supported by more recent evidence (23), which may have further improved their developmental competence. In fact, seasonality and ovarian status are two critical determinants of the developmental potential of domestic-cat oocytes produced by IVP s (39–41). Spindler and Wildt (34) highlighted reduced nuclear maturation and compromised developmental competence during the non-breeding season. To address this, Piras et al. (41) proposed the use of Brilliant Cresyl Blue (BCB) staining to select high-quality oocytes. BCB − oocytes have been shown to exhibit lower mitochondrial activity and reduced reactive oxygen species (ROS) levels after IVM, indicating diminished metabolic function and developmental capacity. This technique may be especially useful for recovering viable oocytes from genetically valuable felids that die during the non-breeding season, thereby enhancing IVP outcomes under suboptimal conditions.

Nevertheless, despite these methodological improvements, the seasonal differences in oocyte yield and overall quality observed in our study suggest that intrinsic physiological factors associated with reproductive seasonality are not entirely mitigated under current IVP conditions. This underscores the complexity of seasonal influences and the continued need for protocol optimization, particularly to improve outcomes from oocytes retrieved during the non-breeding season. From a practical standpoint, our findings suggest that oocyte recovery and *in vitro* embryo production should be preferentially scheduled for late autumn and winter, while additional supportive interventions may be necessary to counteract the physiological limitations encountered during the spring trough.

With respect to donor age, our data reveal a dual profile: ageing diminishes oocyte availability while preferentially permitting those of higher developmental potential to progress, leading to stable, or slightly improved blastocyst production rates in older queens despite lower initial yields. These results are in accordance with previous studies such as bovine (42, 43), in which adult females produced a higher proportion of developmentally competent oocytes capable of reaching the blastocyst stage, thanks to a better maternal RNA storage regulation during IVM. Therefore, in the present study, while blastocyst rates did not differ significantly across seasons, age groups, or recovery methods, biologically relevant trends were evident, like the slight improvement in blastocyst formation in spring. Despite geriatric queens achieving a 34% relative gain over young and adult groups, suggesting a selective retention of high-quality oocytes, the cleaved embryo to blastocyst did not reach statistical significance. No quality analysis of the resulting embryos was included in this study, as performed in other species for assessing effects of seasonality (44), which limits the interpretation of the data and prevents us from drawing definitive conclusions. Recently, Danielson et al. (45), published a study concluding a declining translational capacity of the oocyte. In fact, errors in female meiosis, which increase with age, contribute to embryonic aneuploidy and miscarriage. Oocytes depend on long-lived mRNAs and ribosomes accumulated during growth, making them vulnerable to disruptions in protein synthesis. This reliance may underlie age-related meiotic instability and infertility. Understanding these mechanisms could inform new fertility treatments. In the same line, an insightful study included reciprocal nuclear transfer experiments demonstrating that cytoplasmic, rather than nuclear, factors are responsible for the formation of abnormal meiotic spindles in aged animals, supporting the hypothesis that age-related alterations in translational capacity contribute to spindle instability (46) underlying the importance to include cytoplasmic evaluation of the oocyte. Future work should therefore incorporate high-resolution imaging of mitochondrial distribution and proteomic profiling to discern whether the apparent geriatric advantage is genuine or a consequence of stringent selection.

Analogous interventions in other species have shown promising results. Therefore, and building further on the results about seasonality, in the cases in which oocytes may suffer from deleterious effects from an advanced or pre-pubertal age of the donor. Thus, protocol adjustments may help mitigate age-related oocyte deficiencies. Zingerenko et al. (47), explored that, in women, negative effects existent in oocytes from women in an advanced age, may be reversed with the supplementation of extracellular vesicles isolated from younger individuals. Similarly, in the case of pre-pubertal oocytes in bovine, IVP results were enhanced by the supplementation of melatonin, in contrast to the difference found between samples from adult cows and pre-pubertal individuals without suppplementation (38).

Lastly, the individual and reproductive differences of the queens could not be explored in the present study due to the origin of the samples. However, the influence of estrous cycle stage on *in vitro* embryonic development has been studied in the past in the domestic cat (35), relating the oocytes obtained from a luteal or inactive individuals to a compromised blastocyst rate. This factor may have acted in combination with age and individual characteristics of the donors, producing the variable results described above. Such findings can guide donor selection and timing of oocyte retrieval in both clinical and conservation settings. Our season- and age-tailored IVP guidelines can (i) inform sample-allocation strategies for endangered felids, (ii) refine gamete- and embryo-cryopreservation schedules, and (iii) reduce the number of exploratory cycles required when adapting protocols to small populations.

Collectively, these findings offer valuable insights into the multifactorial nature of IVP success in domestic cats, highlighting the influence of seasonal and physiological variables. They underscore the need for targeted adjustments to culture conditions to optimize outcomes. Continued research in this area will not only advance ARTs in felids but also serve as a foundation for future studies on embryo-maternal communication. Such progress has the potential to transform current empirical approaches into rigorously optimized protocols, enhancing clinical feline reproduction, supporting conservation breeding programs for threatened species, and contributing to broader comparative research in reproductive biomedicine.

## Data Availability

The original contributions presented in the study are included in the article/Supplementary material, further inquiries can be directed to the corresponding author.
