# Supplementary material for: Seasonal and age effects on in vitro embryo production in domestic cats under a standardized protocol
Source: Front Vet Sci. 2025 Aug 19;12:1647305. doi: 10.3389/fvets.2025.1647305 (PMC12404156; doi:10.3389/fvets.2025.1647305)
Supplement: Supplementary file 1 [file Table_1.DOCX]

Supplementary Material

# Supplementary Tables

**Supplementary Table 1.** Seasonal variation in the average number of recovered oocytes and the percentage of selected oocytes per replicate. Values are expressed as mean ± SEM.

| **Season** | **Avg recovered oocytes** | **SEM** | **% selected oocytes** | **SEM** |
| --- | --- | --- | --- | --- |
| Winter | 26.91 | 2.56 | 56.09 | 4.89 |
| Spring | 20.57 | 1.57 | 67.62 | 5.08 |
| Summer | 21.47 | 1.89 | 42.23 | 3.97 |
| Autumn | 25.72 | 2.45 | 55.54 | 4.12 |

**Supplementary Table 2.** Seasonal differences in embryo developmental competence. Data are presented as mean ± SEM for blastocyst rates per zygote and per cleaved embryo.

| **Season** | **Blastocysts/zygotes** | **SEM** | **Blastocysts/cleaved** | **SEM** |
| --- | --- | --- | --- | --- |
| Winter | 15.95 | 3.28 | 26.36 | 4.89 |
| Spring | 9.10 | 3.75 | 26.46 | 5.08 |
| Summer | 8.62 | 3.97 | 17.29 | 3.97 |
| Autumn | 9.46 | 3.78 | 16.86 | 4.12 |

**Supplementary Table 3.** Average number of recovered oocytes and percentage of selected oocytes per replicate across months. Data are shown as mean ± SEM.

| **Month** | **Avg recovered oocytes** | **SEM** | **% selected oocytes** | **SEM** |
| --- | --- | --- | --- | --- |
| January | 27.51 | 2.53 | 56.62 | 3.78 |
| February | 28.38 | 2.67 | 57.91 | 3.23 |
| March | 22.77 | 2.32 | 51.53 | 3.56 |
| April | 15.86 | 1.90 | 54.06 | 2.90 |
| May | 24.55 | 2.25 | 66.35 | 3.45 |
| June | 21.93 | 2.97 | 61.81 | 4.03 |
| July | 20.15 | 2.45 | 42.91 | 3.33 |
| October | 30.63 | 2.04 | 33.69 | 3.88 |
| November | 29.57 | 2.67 | 58.10 | 4.01 |
| December | 20.62 | 2.89 | 53.41 | 2.90 |

**Supplementary Table 4.** Monthly variation in embryo developmental competence. Mean ± SEM values are given for blastocyst rates per zygote and per cleaved embryo.

| **Month** | **Blastocysts/zygotes** | **SEM** | **Blastocysts/cleaved** | **SEM** |
| --- | --- | --- | --- | --- |
| January | 14.86 | 2.99 | 26.30 | 2.19 |
| February | 16.16 | 2.76 | 28.73 | 3.20 |
| March | 15.96 | 2.24 | 21.51 | 2.80 |
| April | 6.67 | 1.73 | 13.99 | 2.29 |
| May | 10.05 | 2.44 | 36.87 | 3.43 |
| June | 13.59 | 1.79 | 35.34 | 3.37 |
| July | 6.43 | 2.41 | 13.63 | 4.82 |
| October | 6.51 | 1.14 | 13.68 | 1.53 |
| November | 15.38 | 1.63 | 25.59 | 2.37 |
| December | 12.59 | 1.71 | 22.85 | 2.78 |

**Supplementary Table 5.** Average number of recovered oocytes and percentage of selected oocytes per replicate in queens of different age groups. Data are presented as mean ± SEM.

| **Age** | **Avg recovered oocytes** | **SEM** | **% selected oocytes** | **SEM** |
| --- | --- | --- | --- | --- |
| Young | 26.86 | 2.89 | 52.39 | 4.23 |
| Adult | 21.29 | 2.45 | 59.31 | 4.89 |
| Geriatric | 17.67 | 3.76 | 57.03 | 4.50 |

**Supplementary Table 6.** Embryo developmental competence by age group. Values represent the mean ± SEM for blastocyst rates per zygote and per cleaved embryo.

| **Age** | **Blastocysts/zygotes** | **SEM** | **Blastocysts/cleaved** | **SEM** |
| --- | --- | --- | --- | --- |
| Young | 12.5 | 1.13 | 23.39 | 2.46 |
| Adult | 11.5 | 1.25 | 24.72 | 1.90 |
| Geriatric | 16.5 | 2.56 | 26.55 | 2.05 |
